# Taxonomic study of the genera *Halogeometricum* and *Halosarcina*: transfer of *Halosarcina limi* and *Halosarcina pallida* to the genus *Halogeometricum* as *Halogeometricum* *limi* comb. nov. and *Halogeometricum* *pallidum* comb. nov., respectively

**DOI:** 10.1099/ijs.0.055038-0

**Published:** 2013-10

**Authors:** Xing-Xing Qiu, Mei-Lin Zhao, Dong Han, Wen-Jiao Zhang, Mike L. Dyall-Smith, Heng-Lin Cui

**Affiliations:** 1 School of Food & Biological Engineering, Jiangsu University, Zhenjiang 212013, People’s Republic of China; 2 School of Biomedical Sciences, Charles Sturt University, Boorooma Street, Wagga Wagga, NSW, 2678 Australia

## Abstract

Members of the haloarchaeal genera *Halosarcina* and *Halogeometricum* (family *Halobacteriaceae*) are closely related to each other and show 96.6–98 % 16S rRNA gene sequence similarity. This is higher than the accepted threshold value (95 %) to separate two genera, and a taxonomic study using a polyphasic approach of all four members of the two genera was conducted to clarify their relationships. Polar lipid profiles indicated that *Halogeometricum rufum* RO1-4^T^, *Halosarcina pallida* BZ256^T^ and *Halosarcina limi* RO1-6^T^ are related more to each other than to *Halogeometricum borinquense* CGMCC 1.6168^T^. Phylogenetic analyses using the sequences of three different genes (16S rRNA gene, *rpoB*′ and *EF-2*) strongly supported the monophyly of these four species, showing that they formed a distinct clade, separate from the related genera *Halopelagius*, *Halobellus*, *Haloquadratum*, *Haloferax* and *Halogranum*. The results indicate that the four species should be assigned to the same genus, and it is proposed that *Halosarcina pallida* and *Halosarcina limi* be transferred to the genus *Halogeometricum* as *Halogeometricum* *pallidum* comb. nov. (type strain, BZ256^T^ = KCTC 4017^T^ = JCM 14848^T^) and *Halogeometricum* *limi* comb. nov. (type strain, RO1-6^T^ = CGMCC 1.8711^T^ = JCM 16054^T^).

The genus *Halogeometricum* was proposed in 1998 to accommodate a pleomorphic strain, *Halogeometricum borinquense* PR3^T^, isolated from the solar salterns of Cabo Rojo, Puerto Rico (Montalvo-Rodríguez *et al.*, 1998). The phenotypic features and its phylogenetic position indicated that it was distinct from related genera, and a major glycolipid GLb (Cui *et al.*, 2010c) detected in *Halogeometricum borinquense* PR3^T^ served as the characteristic glycolipid for describing this genus. In 2008, the novel genus *Halosarcina* was proposed to accommodate a sarcina-shaped strain, BZ256^T^, that showed 96.8 % 16S rRNA gene sequence similarity to *Halogeometricum borinquense* PR3^T^ and contained a major glycolipid (S-DGD-1) that was chromatographically different from GLb (Savage *et al.*, 2008; Cui *et al.*, 2010b). The species *Halogeometricum rufum* RO1-4^T^ and *Halosarcina limi* RO1-6^T^ were described two years later, and emended descriptions of the genera *Halogeometricum* and *Halosarcina*, including the polar lipid profiles, were reported (Cui *et al.*, 2010b; Cui *et al.*, 2010c). However, the four members of the genera *Halosarcina* and *Halogeometricum* are closely related to each other, showing 96.6–98 % 16S rRNA gene sequence identity, a level that is higher than the ‘lower cut-off’ value (95 %) reported by Yarza *et al.*, (2008) and that generally marks genus-level boundaries in prokaryotes. To elucidate the taxonomy of these genera, the type strains *Halogeometricum borinquense* CGMCC 1.6168^T^, *Halogeometricum rufum* RO1-4^T^, *Halosarcina pallida* BZ256^T^ and *Halosarcina limi* RO1-6^T^ were directly compared in a polyphasic taxonomic study.

The strains were routinely grown aerobically at 37 °C in NOM medium (Cui *et al.*, 2011a). Phenotypic tests were performed according to the proposed minimal standards for description of new taxa in the order *Halobacteriales* (Oren *et al.*, 1997). *Halobacterium jilantaiense* NG4^T^, *Haloferax volcanii* CGMCC 1.2150^T^ and *Haloarcula marismortui* CGMCC 1.1784^T^ were used as reference strains. Various tests relating to morphology and growth characteristics, nutrition, miscellaneous biochemical activities and sensitivity to antimicrobial agents were performed as described previously (Cui *et al.*, 2012).

Other than the type strains of genera *Halogeometricum* and *Halosarcina*, three other strains belonging to the *Halogeometricum*/*Halosarcina* cluster, strains RO3-11, HO1-4 and GSL-24, were also included in the analysis of polar lipid composition. Polar lipids were extracted using a chloroform/methanol system and analysed using one- and two-dimensional TLC, as described previously (Cui *et al.*, 2010a). Merck silica gel 60 F_254_ aluminium-backed thin-layer plates were used in TLC analysis. In two-dimensional TLC, the first solvent was chloroform/methanol/water (65 : 25 : 4, by vol.) and the second solvent was chloroform/methanol/acetic acid/water (80 : 12 : 15 : 4, by vol.). The latter solvent was also used in one-dimensional TLC. Two specific detection spray reagents were used; phosphate stain reagent for phospholipids and α-naphthol stain for glycolipids. The general detection reagent, sulfuric acid/ethanol (1 : 2, v/v) was used to detect total polar lipids.

Genomic DNAs from halophilic archaeal strains were prepared as described previously (Cui *et al.*, 2011b). The 16S rRNA genes were amplified, cloned and sequenced according to a previously described protocol (Cui *et al.*, 2009). PCR-mediated amplification and sequencing of the *rpoB′* genes were carried out as described previously (Minegishi *et al.*, 2010). The *EF-2* genes were amplified and sequenced using the primer pair EF-2f (5′-ATGGGYMGACGHAAGAA-3′) and EF-2r (5′-GCBGGRCCRCGGTGGAT-3′). These primers were designed (this study) using aligned genomic sequences encoding the *EF-2* genes from 26 genera of the family *Halobacteriaceae* (sequences downloaded from the GenBank database). Multiple sequence alignments were performed using the clustal w program integrated in the mega 5 software (http://www.megasoftware.net/). Phylogenetic trees were reconstructed using the neighbour-joining, maximum-parsimony (MP) and maximum-likelihood (ML) algorithms in the mega 5 software. Gene sequence similarity values were calculated using the pairwise-distance computing function of mega 5.

*Halogeometricum borinquense* CGMCC 1.6168^T^, *Halogeometricum rufum* RO1-4^T^, *Halosarcina pallida* BZ256^T^ and *Halosarcina limi* RO1-6^T^ shared similar phenotypic features. They stained Gram-negative, required Mg^2+^ for growth, grew best at mesophilic temperatures (25–45 °C) and over the pH range 6–8. They did not hydrolyse starch, did not produce H_2_S from sodium thiosulfate, were sensitive novobiocin, bacitracin, rifampicin, mycostatin and nitrofurantoin and resistant to trimethoprim, erythromycin, ampicillin, penicillin G, chloramphenicol, neomycin, ciprofloxacin, streptomycin, kanamycin, vancomycin, norfloxacin, tetracycline, gentamicin and nalidixic acid. They utilized d-glucose, d-galactose, sucrose, glycerol, acetate, pyruvate, l-alanine and l-glutamate, but did not use d-ribose, d-mannitol, citrate, l-aspartate or l-ornithine.

The main phenotypic characteristics differentiating the four species from each other were: cell shape, motility, colony colour, optimum NaCl, optimum Mg^2+^, growth temperature and pH, anaerobic growth with nitrate, gas formation from nitrate, nitrate reduction, indole formation, casein hydrolysis, gelatin liquefaction and utilization of specific carbon sources (Table 1). The differential phenotypic characteristics of the four species clearly distinguish between them at the species level but they are not helpful regarding genus-level relationships.

**Table 1.** Characteristics that differentiate *Halogeometricum rufum* RO1-4^T^, *Halogeometricum borinquense* JCM 10706^T^, *Halosarcina pallida* BZ256^T^ and *Halosarcina limi* RO1-6^T^ Taxa: 1, *Halogeometricum borinquense* JCM 10706^T^; 2, *Halogeometricum rufum* RO1-4^T^; 3, *Halosarcina pallida* BZ256^T^; 4, *Halosarcina limi* RO1-6^T^. +, Positive; −, negative.

Characteristic	1	2	3	4
Cell shape	Pleomorphic	Pleomorphic	Coccus	Pleomorphic
Motility	+	+	−	+
Colony colour	Pink	Red	Pink	Red
Optimum NaCl (M)	3.4–4.3	3.9	3.1	3.9
Optimum Mg^2+^(M)	0.04–0.08	0.3	0.1–0.3	0.3
Optimum temperature (°C)	40	40–42	30	37
Optimum pH	7.0	7.0	6.5	7.0
Anaerobic growth with nitrate	+	−	−	−
Gas formation from nitrate	+	−	−	−
Nitrate reduction	+	+	−	+
Indole formation	+	+	+	−
Casein hydrolysis	+	−	−	−
Gelatin liquefaction	+	−	−	−
Utilization of:				
d-Mannose	+	+	−	+
d-Fructose	+	−	−	−
d-Xylose	+	−	−	−
Maltose	+	+	+	−
Lactose	+	+	−	+
dl-Lactate	+	+	−	+
DNA G+C content (mol%)	59.1	64.9	65.4	61.2

The lipids of all strains were examined by one- and two-dimensional TLC, and the results are presented in Fig. S1 and summarized in Table S1 available in IJSEM Online. The common polar lipids phosphatidylglycerol and phosphatidylglycerol phosphate methyl ester were found in all four strains, as were glycolipids GL1 and GL3. Three other glycolipids (GL2, GL4 and GL5) were present in three of the strains (*Halogeometricum rufum* RO1-4^T^, *Halosarcina pallida* BZ256^T^ and *Halosarcina limi* RO1-6^T^) but not in *Halogeometricum borinquense* CGMCC 1.6168^T^. The glycolipid GLb is the major polar lipid of *Halogeometricum borinquense* CGMCC 1.6168^T^ [Fig. S1(i,iii)] and a chromatographically identical lipid was detected (at low levels) in two other strains [*Halogeometricum rufum* RO1-4^T^ and *Halosarcina limi* RO1-6^T^: Fig S1(ii)]. Two other lipids, P1 and P2, are distributed in an overlapping pattern across the strains, with *Halogeometricum rufum* RO1-4^T^ having both lipids, while each of the other strains has only one of them. The polar lipids profiles of strains RO3-11, HO1-4 and GSL-24 are similar to that of *Halosarcina pallida* BZ256^T^ [Fig. S1(iv)]. The summary table (Table S1) more clearly indicates those lipids shared by all strains (GL1 and GL3), those shared by three of the strains (GLb, GL2, GL4, GL5 and P1), or only two of the strains (P2). The shared lipids observed in these strains would be consistent with them being members of the same genus, although phylogenetic methods are needed to accurately determine this.

The 16S rRNA gene sequence similarities of the seven species ranged from 96.4 % to 99.5 %. All of these values are above the recently described threshold of 95 % for delineating prokaryotic genera (Yarza *et al.*, 2008; Tindall *et al.*, 2010). Phylogenetic tree reconstructions based on 16S rRNA gene sequences revealed that these species formed a tight cluster, with high bootstrap confidence and were distinct from the related genera, *Halobellus*, *Haloquadratum*, *Halopelagius* and *Haloferax* (Fig. 1a). The members of the genera *Halogeometricum* and *Halosarcina* did not branch as distinct monophyletic clades related to their currently assigned genera, but specifically clustered together as a paraphyletic group, indicating that these taxa are phylogenetically related at the genus level. This phylogenetic position was also supported by tree reconstructions generated using the MP and ML algorithms (not shown).

**Fig. 1.**
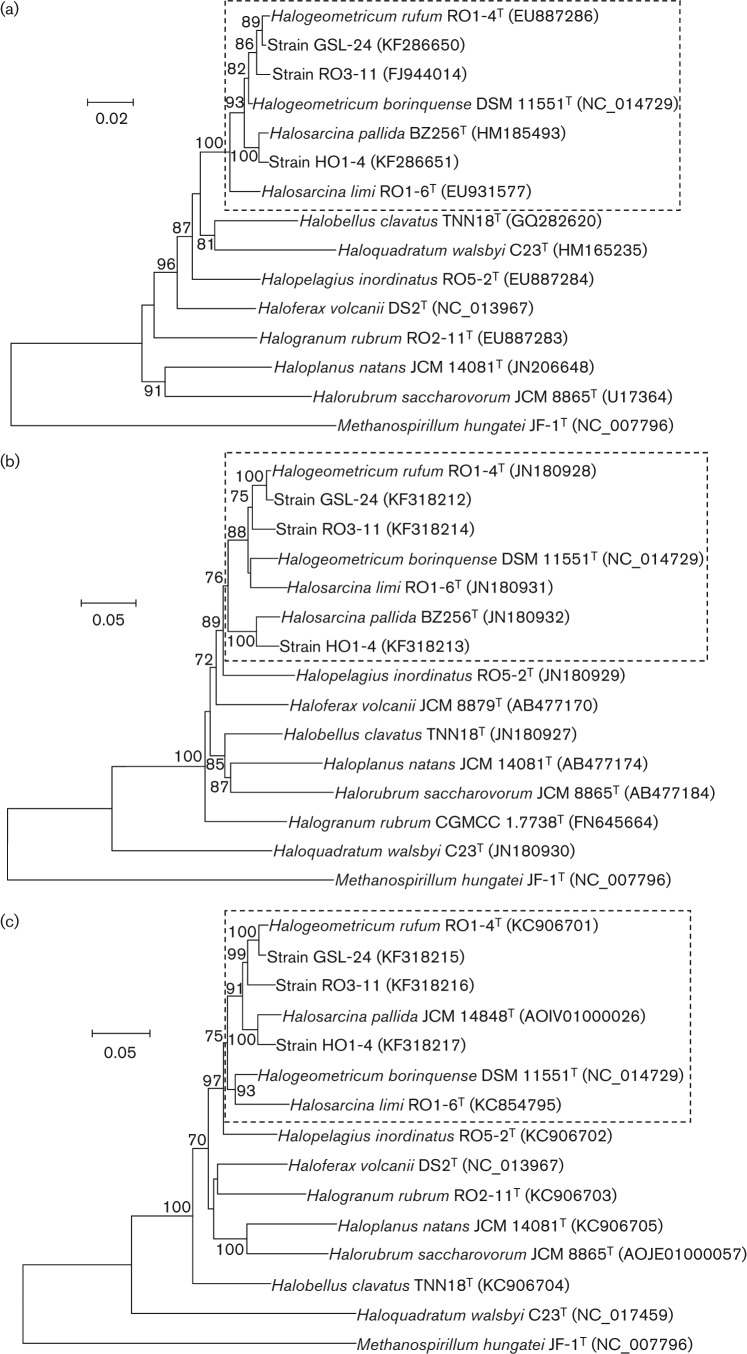
Neighbour-joining phylogenetic tree reconstructions based on 16S rRNA gene sequences (a), *rpoB′* gene sequences (b) and *EF-2* gene sequences (c) showing the relationships between members of the genera *Halogeometricum*, *Halosarcina* and related genera within the family *Halobacteriaceae*. Bootstrap values (%) are based on 1000 replicates and are shown for branches with >70 % bootstrap support. Dashed lines enclose clades that are the members of the genera *Halogeometricum* and *Halosarcina*. Bars, 0.02 (a) and 0.05 (b, c) substitutions per nucleotide position.

A recent taxonomic study of the *Halobacteriaceae* has proposed that a similarity value less than 86.2 % between *rpoB′* genes can be used to distinguish genera (Minegishi *et al.*, 2010). The *rpoB*′ genes of the four type strains, *Halogeometricum borinquense* CGMCC 1.6168^T^, *Halogeometricum rufum* RO1-4^T^, *Halosarcina pallida* BZ256^T^ and *Halosarcina limi* RO1-6^T^ and strains RO3-11, HO1-4 and GSL-24 were sequenced and found to be identical in length 1833 bp and showed 88.5–99.0 % identity to each other. This level of similarity is higher than the genus-level threshold recommended by Minegishi *et al.*, (2010). Phylogenetic tree reconstructions based on *rpoB*′ genes revealed that the four species clustered tightly together (at high bootstrap confidence) and were separate from the related genera *Halopelagius*, *Haloferax* and *Halobellus* (Fig. 1b). Similar to the 16S rRNA gene sequence trees, these results support the view that all four species belong to the same genus. Trees generated using the MP and ML algorithms gave similar results (not shown).

The housekeeping gene *EF-2* encodes translation elongation factor EF-2 and has been shown to be useful in taxonomy of *Halobacteriaceae* (Papke *et al.*, 2011; Oren, 2012). This gene (about 2190 nt) was amplified from all seven strains using the primers designed in this study. The sequenced genes were found to be 90.1–98.8 % identical, and phylogenetic analysis revealed that the four species formed a monophyletic clade (high bootstrap confidence), separate from the related genera, *Halopelagius*, *Haloferax* and *Halogranum* (Fig. 1c). MP and ML algorithms gave similar results (data not shown).

This polyphasic taxonomic study has provided clear evidence that the four species are sufficiently related that they should belong to the same genus. On the basis of these results, we propose that *Halosarcina pallida* and *Halosarcina limi* be transferred to the genus *Halogeometricum* as *Halogeometricum* *pallidum* comb. nov. (type strain, BZ256^T^ = KCTC 4017^T^ = JCM 14848^T^) and *Halogeometricum* *limi* comb. nov. (type strain, RO1-6^T^ = CGMCC 1.8711^T^ = JCM 16054^T^).

## Description of *Halogeometricum* *limi* comb. nov.

*Halogeometricum* *limi* (li′mi. L. gen. n. *limi* of/from mud).

Basonym: *Halosarcina limi* Cui *et al.* 2010.

The description is identical to that of *Halosarcina limi* given by Cui *et al.* (2010b). The type strain is RO1-6^T^ ( = CGMCC 1.8711^T^ = JCM 16054^T^).

## Description of *Halogeometricum* *pallidum* comb. nov.

*Halogeometricum* *pallidum* (pal′li.dum. L. neut. adj. *pallidum* pale).

Basonym: *Halosarcina pallida* Savage *et al.* 2008.

The description is identical to that of *Halosarcina pallida* given by Savage *et al.* (2008). The type strain is BZ256^T^ ( = KCTC 4017^T^ = JCM 14848^T^).

## References

[r1] Cui H.-L. Zhou P.-J. Oren A. Liu S.-J. **(**2009**).** Intraspecific polymorphism of 16S rRNA genes in two halophilic archaeal genera, *Haloarcula* and *Halomicrobium*. Extremophiles 13, 31–37 10.1007/s00792-008-0194-2 18836684

[r2] Cui H.-L. Gao X. Yang X. Xu X.-W. **(**2010a**).** *Halorussus rarus* gen. nov., sp. nov., a new member of the family *Halobacteriaceae* isolated from a marine solar saltern. Extremophiles 14, 493–499 10.1007/s00792-010-0329-0 20824294

[r3] Cui H.-L. Gao X. Li X.-Y. Xu X.-W. Zhou Y.-G. Liu H.-C. Zhou P.-J. **(**2010b**).** *Halosarcina limi* sp. nov., a halophilic archaeon from a marine solar saltern, and emended description of the genus *Halosarcina*. Int J Syst Evol Microbiol 60, 2462–3466 10.1099/ijs.0.018697-0 19946053

[r4] Cui H.-L. Yang X. Gao X. Li X.-Y. Xu X.-W. Zhou Y.-G. Liu H.-C. Zhou P.-J. **(**2010c**).** *Halogeometricum rufum* sp. nov., a halophilic archaeon from a marine solar saltern, and emended description of the genus *Halogeometricum*. Int J Syst Evol Microbiol 60, 2613–2617 10.1099/ijs.0.019463-0 20023063

[r5] Cui H.-L. Gao X. Yang X. Xu X.-W. **(**2011a**).** *Halolamina pelagica* gen. nov., sp. nov., a new member of the family *Halobacteriaceae*. Int J Syst Evol Microbiol 61, 1617–1621 10.1099/ijs.0.026799-0 20693359

[r6] Cui H.-L. Yang X. Mou Y.-Z. **(**2011b**).** *Salinarchaeum laminariae* gen. nov., sp. nov.: a new member of the family *Halobacteriaceae* isolated from salted brown alga *Laminaria*. Extremophiles 15, 625–631 10.1007/s00792-011-0393-0 21901373

[r7] Cui H.-L. Mou Y.-Z. Yang X. Zhou Y.-G. Liu H.-C. Zhou P.-J. **(**2012**).** *Halorubellus salinus* gen. nov., sp. nov. and *Halorubellus litoreus* sp. nov., novel halophilic archaea isolated from a marine solar saltern. Syst Appl Microbiol 35, 30–34 10.1016/j.syapm.2011.08.001 21889861

[r8] Minegishi H. Kamekura M. Itoh T. Echigo A. Usami R. Hashimoto T. **(**2010**).** Further refinement of the phylogeny of the *Halobacteriaceae* based on the full-length RNA polymerase subunit B′ (*rpoB*^′^) gene. Int J Syst Evol Microbiol 60, 2398–2408 10.1099/ijs.0.017160-0 19946058

[r9] Montalvo-Rodríguez R. Vreeland R. H. Oren A. Kessel M. Betancourt C. López-Garriga J. **(**1998**).** *Halogeometricum borinquense* gen. nov., sp. nov., a novel halophilic archaeon from Puerto Rico. Int J Syst Bacteriol 48, 1305–1312 10.1099/00207713-48-4-1305 9828431

[r10] Oren A. **(**2012**).** Taxonomy of the family *Halobacteriaceae*: a paradigm for changing concepts in prokaryote systematics. Int J Syst Evol Microbiol 62, 263–271 10.1099/ijs.0.038653-0 22155757

[r11] Oren A. Ventosa A. Grant W. D. **(**1997**).** Proposed minimal standards for description of new taxa in the order *Halobacteriales*. Int J Syst Bacteriol 47, 233–238 10.1099/00207713-47-1-233

[r12] Papke R. T. White E. Reddy P. Weigel G. Kamekura M. Minegishi H. Usami R. Ventosa A. **(**2011**).** A multilocus sequence analysis approach to the phylogeny and taxonomy of the *Halobacteriales*. Int J Syst Evol Microbiol 61, 2984–2995 10.1099/ijs.0.029298-0 21296924

[r13] Savage K. N. Krumholz L. R. Oren A. Elshahed M. S. **(**2008**).** *Halosarcina pallida* gen. nov., sp. nov., a halophilic archaeon from a low-salt, sulfide-rich spring. Int J Syst Evol Microbiol 58, 856–860 10.1099/ijs.0.65398-0 18398182

[r14] Tindall B. J. Rosselló-Móra R. Busse H. J. Ludwig W. Kämpfer P. **(**2010**).** Notes on the characterization of prokaryote strains for taxonomic purposes. Int J Syst Evol Microbiol 60, 249–266 10.1099/ijs.0.016949-0 19700448

[r15] Yarza P. Richter M. Peplies J. Euzéby J. Amann R. Schleifer K. H. Ludwig W. Glöckner F. O. Rosselló-Móra R. **(**2008**).** The All-Species Living Tree project: a 16S rRNA-based phylogenetic tree of all sequenced type strains. Syst Appl Microbiol 31, 241–250 10.1016/j.syapm.2008.07.001 18692976

